# Genomic breeding value prediction and QTL mapping of QTLMAS2010 data using Bayesian Methods

**DOI:** 10.1186/1753-6561-5-S3-S13

**Published:** 2011-05-27

**Authors:** Xiaochen Sun, David Habier, Rohan L Fernando, Dorian J Garrick, Jack CM Dekkers

**Affiliations:** 1 Department of Animal Science and Center for Integrated Animal Genomics, Iowa State University, Ames, Iowa 50011, USA

## Abstract

**Background:**

Bayesian methods allow prediction of genomic breeding values (GEBVs) using high-density single nucleotide polymorphisms (SNPs) covering the whole genome with effective shrinkage of SNP effects using appropriate priors. In this study we applied a modification of the well-known BayesA and BayesB methods to estimate the proportion of SNPs with zero effects (π) and a common variance for non-zero effects. The method, termed BayesCπ, was used to predict the GEBVs of the last generation of the QTLMAS2010 data. The accuracy of GEBVs from various methods was estimated by the correlation with phenotypes in the last generation. The methods were BayesCPi and BayesB with different π values, both with and without polygenic effects, and best linear unbiased prediction using an animal model with a genomic or numerator relationship matrix. Positions of quantitative trait loci (QTLs) were identified based on the variances of GEBVs for windows of 10 consecutive SNPs. We also proposed a novel approach to set significance thresholds for claiming QTL in this specific case by using pedigree-based simulation of genotypes. All analyses were focused on detecting and evaluating QTL with additive effects.

**Results:**

The accuracy of GEBVs was highest for BayesCπ, but the accuracy of BayesB with π equal to 0.99 was similar to that of BayesCπ. The accuracy of BayesB dropped with a decrease in π. Including polygenic effects into the model only had marginal effects on accuracy and bias of predictions. The number of QTL identified was 15 when based on a stringent 10% chromosome-wise threshold and increased to 21 when a 20% chromosome-wise threshold was used.

**Conclusions:**

The BayesCπ method without polygenic effects was identified to be the best method for the QTLMAS2010 dataset, because it had highest accuracy and least bias. The significance criterion based on variance of 10-SNP windows allowed detection of more than half of the QTL, with few false positives.

## Background

Genomic prediction of breeding values of individuals is based on a large number of SNPs across the whole genome giving high-density coverage. Each QTL is expected to be in linkage disequilibrium (LD) with at least one SNP because of the high marker density, hence the effects of all QTL are expected to be captured by SNPs [1]. Bayesian methods enable prediction of the effects of high-density SNPs covering the whole genome, even when the number of SNPs is much larger than the number of individuals with phenotypic and genotypic records. By specifying proper prior distributions for SNP effects, the ignorable small SNP effects are coerced to zero and only SNPs with larger effects on phenotype are fitted in the model. In BayesB, as proposed by Meuwissen *et al.*[1], the prior specification for a SNP effect is zero with fixed probability π, and normally distributed with a locus-specific variance with probability (1-π). The variance has an inverted Chi-square distribution with known degrees of freedom and scale parameter derived from the assumed known additive genetic variance. In this study, we applied a modification of BayesB, BayesCπ [2], where a single effect variance is common to all SNPs with non-zero effects, and the probability that a SNP has zero effect, π, is treated as unknown. This modification aims at overcoming the drawbacks of BayesB pointed out by Gianola *et al.*[*3*], that the full-conditional posterior distribution is dominated by the prior and not by the data.

The availability of genome-wide SNP panels enables detection of statistical associations between a trait and any SNP in terms of a genome-wide association study (GWAS), enhancing the possibility of mapping QTL across the genome [4]. Bayesian methods, such as those described above, are useful for GWAS QTL mapping because the inferences are based on the joint posterior distribution, which takes full account of all unknown parameters [5,6]. The posterior probability of inclusion of each SNP into the model (we will refer to this as the model frequency) is mostly used as the criterion to detect QTL [7], as well as its derivatives, such as the Bayes factor [7], estimated QTL intensity [8], and the Bayes information criterion [9]. Theoretically, within a class of SNPs that have the same model frequency, the model frequency indicates the proportion of the SNPs among them that are associated with QTL. This is, however, not always the true, especially when QTL and SNPs are in high LD and the effect of a single QTL could be spread over multiple SNPs. Therefore, to address the problem of model frequency with high density SNP panels, new criteria are needed to claim presence of a QTL in a frequentist way. Permutation tests, such as those used in least squares or maximum likelihood QTL interval mapping [10] for cross or family designs are not possible when data are from complex pedigrees, as was the case for the QTLMAS 2010 data.

Against this background, in this study we aimed to: (i) identify the Bayesian approach that most accurately predicts GEBV for the QTLMAS2010 data; (ii) develop a new criterion based on the 10-SNP window variance for QTL detection to concentrate signals from high density SNP panels; and (iii) set significance thresholds for the window variance criterion to claim QTL when pedigree relationships exist among individuals.

## Methods

### Dataset

The simulated dataset was provided in advance of the 14^th^ European QTL-MAS Workshop [11]. The population consisted of individuals in 5 generations (including founders) from 20 founders. Individuals from the first four generations had phenotypes for a quantitative trait. Full pedigree and gender were known. The genome contained 5 chromosomes, each 100 million base-pairs in length. All individuals were genotyped for 10,031 SNPs that were evenly spaced across the genome.

### Predicting GEBVs

Four methods were used and compared for estimation of the marker effects and GEBV: BayesB [1], BayesCπ [2], an animal model using the genomic relationship matrix (G-BLUP), and an animal model using the numerator-relationship matrix (P-BLUP). The latter P-BLUP results in the standard pedigree-based BLUP EBVs [12]. The effect of including polygenic effects was also investigated for the marker-based methods (BayesB, BayesCπ and G-BLUP). The statistical model for the marker-based methods with polygenic effects was

where *y* is an *N* × 1 vector of phenotypes with *N* being the numbers of individuals, *μ* is the overall mean, W is the incidence matrix for gender, *s* is a 2 × 1 vector with fixed gender effects, *u* is a vector with random polygenic effects of all individuals with , ( A is the numerator relationship matrix and  is the polygenic variance), X*_j_* is an *N* × 1 vector of genotypes at SNP *j* , coded , *α_j_* is the random allele substitution effect for SNP *j*, *δj* is a 0/1-indicator variable which equals 1 if SNP *j* is included in the model and zero otherwise, and *e* is a vector of random residuals. Given the estimated marker effects and marker genotypes of an individual, its GEBV was calculated by

where  is the estimated polygenic effect of individual *i*, X*_ij_* is the marker genotype at SNP *j* of individual *i*, and  is the estimated effect of SNP *j*.

Method G-BLUP fitted all SNPs in the model, assuming that every SNP explained an equal proportion of the total genetic variance. Model BayesCπ was a modification of model BayesB of Meuwissen *et al*. [1], and was described in detail by Habier *et al*. [2]. Model BayesCπ differs from BayesA and BayesB in its specification of the probability that a SNP has zero effect (π) and the variance of SNP effects (). In BayesA and BayesB, each SNP has a locus-specific effect variance and this variance has a scaled inverted Chi-square distribution with degrees of freedom *v_a_* and scale , which are functions of the assumed known additive genetic variance [1]. In BayesCπ, all SNP effects (α_j_) have a common variance, i.e.  , which has a scaled inverse Chi-square prior distribution with degrees of freedom v_a_ and scale . As a result, the marginal distribution of all SNP effects in BayesCπ is a multivariate student’s t-distribution, [2]. Furthermore, in BayesCπ the probability that a SNP has zero effect (π) was treated as unknown with uniform (0, 1) prior. The prior for residuals e was a normal distribution with mean 0 and variance . Gibbs sampling was applied to calculate the posterior means of model parameters , and π. The MCMC algorithms were run for 50,000 samples, with the first 20,000 samples discarded as burn in.

Effects of SNPs were estimated using the phenotypes and genotypes of individuals in the first three generations (training), which were then used to predict GEBVs of individuals in the fourth generation (validation) to evaluate the accuracy of GEBVs of the marker-based methods. The method giving the highest correlation of GEBVs with phenotypes in the validation population was used to predict the GEBVs of the fifth generation, for which only SNP genotypes but no phenotypes were available. For the fifth generation predictions, the first four generations were used to estimate SNP effects.

### Detecting QTL

The parameter that was used for QTL detection was the variance of the GEBV of chromosome segments comprised of 10 adjacent SNPs, which we termed windows. First, SNP effects and variances were estimated using individuals in the first four generations by BayesCπ, as described above. The GEBV for the 10-SNP window *l* of individual *i*) was computed as

and the variance of this prediction was calculated across individuals in the first four generations. For 1-SNP windows, this method is equivalent to calculating SNP variance as [13] for SNP_j_ . Windows with variance of GEBVs above a predefined threshold were identified as QTL regions. Significant windows that overlapped were considered to identify the same QTL if there was only one variance peak among the SNPs covered by them. The variance for each window was graphically presented against genomic location of the SNP on the x-axis. Within each selected region, the SNP with the largest variance was used to quantify the position and variance of the QTL.

The threshold for the window variance for declaring presence of a QTL was determined by deriving the distribution of the window variance in data simulated under the null hypothesis of no LD between QTL and SNPs. Three strategies were used to generate data sets without LD between QTL and SNPs but using the original phenotypes, so as to maintain the distribution of phenotypes. The first strategy was to simply permute phenotypes against SNP genotypes across individuals in the training data. This strategy maintains LD relationships among SNPs in the original data but breaks all pedigree relationships and prevents SNPs to account for polygenic effects in the permuted data, in contrast to what happens in real data from pedigree populations [14]. The second strategy was to randomly simulate SNP genotypes of individuals in the first 4 generations using the pedigree and SNP placement from the QTLMAS2010 data. The SNPs were assumed to be in linkage equilibrium (LE) in the founder generation. In this case, when estimating the simulated SNP effects using real phenotypes, the SNPs are expected to only capture polygenic effects through the pedigree but not the effects of QTL that underly the existing phenotypes. The simulation assumed that 1 million base-pairs mapped to 1 centimorgan and hence each chromosome was 1 Morgan in length. The third strategy was to simulate LD between the simulated SNPs in the founder generation at a level similar to that found in the QTLMAS2010 data, which was estimated using [15]. Multiple historical generations prior to the founder generation of the pedigree were simulated to create this LD. The effective size (*N_e_*) of the base population was set to 500, and randomly mated for 1,000 discrete generations, then reduced to an effective size of 100, and then increased over the next 10 generations to a size of 1,500, from which the 20 founders of the pedigree were randomly sampled. For all three datasets simulated under the null hypothesis, SNP effects and window variances were estimated using the simulated marker genotypes and real phenotypes by BayesC without polygenic effects and π set equal to the posterior mean of π from BayesCπ when training on the first four generations using the original genotypes, i.e. the method used to obtain GEBVs for the final generation. The latter was done because estimates of π in the simulated null data set were much lower and resulted in very low significance thresholds because variances explained by each SNP were very low. The variances of GEBVs of all 10-SNP windows were calculated using the estimated SNP effects from the simulated data to obtain the distribution of the window variance under the null hypothesis.

To account for multiple testing across a chromosome, significance levels for the window variance were adjusted by dividing desired comparison-wise type I error rates by the effective number of loci (*M_e_*) in the genome, which was calculated by , where *N_e_* is the effective population size and *L* is the length of a chromosome, which was set to 1 Morgan [16]. This can be referred to as a Bonferroni adjustment for multiple testing across each chromosome. To set the thresholds, 10% (primary list) and 20% (secondary list) chromosome-wise type-I error rates were used, where the former was stringent and the latter more liberal.

## Results and discussion

### Accuracy of GEBV prediction

The accuracy of GEBVs was estimated in three ways: (i) the correlation of GEBVs with phenotypes divided by the square root of heritability (estimated from the full dataset with pedigree relationships using ASREML [17], which resulted in ), (ii) the correlation of GEBVs with true breeding values (TBV), and (iii) the correlation of GEBVs with genotypic values. All these three accuracies were based on training on the first 3 generations and validation in generation 4. Results are in Table 1.

**Table 1:** Prediction accuracy of GEBV, correlation of GEBV with TBV, correlation of GEBV with genotypic value (*g*), regression coefficient of phenotype (*y*) on GEBV, regression coefficient of TBV on GEBV, and regression coefficient of genotypic value on GEBV.

Methods	Correlation of GEBV with			Regression coefficient on GEBV of		
	y*^1^	TBV	*g*	*y*	TBV	*g*
**P-BLUP**	0.545	0.410	0.538	1.156	1.003	1.005
**G-BLUP**						
No Poly	0.746	0.610	0.753	1.006	0.949	0.895
Poly	0.737	0.597	0.752	0.961	0.898	0.863
**BayesB, π = 0.75**						
No Poly	0.781	0.632	0.776	1.018	0.950	0.892
Poly	0.778	0.628	0.783	0.984	0.916	0.873
**BayesB, π = 0.95**						
No Poly	0.788	0.640	0.787	1.023	0.960	0.901
Poly	0.784	0.634	0.793	0.983	0.916	0.875
**BayesB, π = 0.99**						
No Poly	0.793	0.646	0.795	1.031	0.967	0.909
Poly	0.790	0.636	0.797	0.981	0.911	0.872
**BayesCπ**						
No Poly	0.796	0.650	0.800	1.011	0.952	0.895
Poly	0.796	0.642	0.804	0.989	0.921	0.880
**BayesCπ gen 5**^2^						
No Poly	–	0.679	0.894	–	0.959	0.965

Results are based on training on the first three generations and validation on generation 4 using P-BLUP, G-BLUP, BayesB with different π's, and BayesCπ, and without (No Poly) and with (Poly) polygenic effects.

^1^Calculated as correlation of phenotype (y) with GEBV, divided by the square root of estimated heritability.

^2^Training on the first 4 generations and predicting generation 5.

The simulated QTLMAS2010 dataset had 30 biallelic additive QTL, 2 pairs of epistatic QTL and 3 paternally imprinted QTL. The QTL from each pair of epistatic QTL were close together and behaved as a single multi-allelic additive QTL. Each of the epistatic QTL-pairs and the imprinted QTL had the same effect as the largest additive QTL. The genotypic value of an individual was the sum of the genotypic value expressed in the phenotype at each of the QTL but the TBV also accounted for the imprinting effects that the individual had on its progeny. Thus, the TBV could deviate considerably from the genotypic values because the imprinted QTLs had large effects. In this study, all marker-based methods only fitted additive effects of SNPs derived based on the regression of SNP genotype on phenotype, which includes the effect of the imprinted QTL. As a result, as shown in Table 1, the accuracy of prediction estimated from the correlation of GEBV with phenotype in the validation population was similar to the correlation of GEBV with genotypic values and the correlation of GEBV with TBV was much lower since the GEBV did not account for imprinting effects of parents on progeny.

The accuracy of P-BLUP was lowest among all methods, as expected. Method G-BLUP, which always fitted all SNPs in the model, had lower accuracy than BayesB and BayesCπ. The Bayesian methods had quite similar accuracies, but BayesCπ tended to be the most accurate. Methods that fitted fewer SNPs performed better than those that fitted more. This might be explained by the fact that under the marker density of QTLMAS2010 data (measured as average *r*^2^=0.22 between adjacent markers on chromosome 1, following Calus *et al.*[18]), there were up to 100 SNPs in strong LD with the QTL and fitting more SNPs in the model resulted in underestimation of the effects of those SNPs.

The posterior mean of π in BayesCπ was 0.988, that is, on average 124 SNPs were fitted in the model, which was similar to that of BayesB when π = 0.99 (Table 2). Also BayesB with π = 0.99 and BayesCπ fitted almost the same subset of SNPs when looking at the model frequencies of the SNPs fitted in the model. This explains the similar accuracy of BayesB with π = 0.99 and BayesCπ.

**Table 2:** Average number of SNPs (#SNP) fitted in the model, estimated variance components, and estimated heritability (Heritability).

Methods	#SNP	Estimated variance components					Heritability
		Marker	Polygenic	Genetic^1^	Residual	Total	

**True value**^2^		–	–	51.76	51.76	103.52	0.500
**P-BLUP**	–	–	54.44	54.44	48.68	103.12	0.528
**G-BLUP**	10031						
No Poly		44.54	–	44.54	54.84	99.38	0.448
Poly		38.53	12.09	50.62	49.04	99.66	0.508
**BayesB, π = 0.75**	2508						
No Poly		44.28	–	44.28	54.08	98.36	0.450
Poly		39.05	11.06	50.11	48.32	98.43	0.509
**BayesB, π = 0.95**	502						
No Poly		43.96	–	43.96	54.16	98.12	0.448
Poly		38.05	12.80	50.85	47.59	98.44	0.517
**BayesB, π = 0.99**	100						
No Poly		43.44	–	43.44	54.58	98.02	0.443
Poly		37.43	12.35	49.78	48.30	98.09	0.508
**BayesCπ**							
No Poly	124	45.68	–	45.68	53.63	99.31	0.460
Poly	80	40.21	10.33	50.54	48.58	99.12	0.510
**BayesCπ gen 5**^3^							
No Poly	92	47.13	–	47.13	53.48	100.61	0.468

Results are based on training on the first three generations and validation on generation 4 using P-BLUP, G-BLUP, BayesB with different π’s, and BayesCπ, and without (No Poly) and with (Poly) polygenic effects.

^1^Total genetic variance = marker variance + polygenic variance.

^2^Total QTL variance = residual variance = 51.76 in the QTLMAS2010 dataset.

^3^Training on the first 4 generations.

The bias of GEBV was evaluated based on the departure from unity of the regression coefficients of phenotype, TBV, and genotypic value on GEBV in the validation data (Table 1). In general, all regression coefficients were very close to 1, showing that biases were small for all methods. For the marker-based methods, the regression coefficients of phenotype on GEBV were closest to 1; regression coefficients for TBV and genetic value were less than 1 and tended to be smallest for genotypic value. All regression coefficients dropped when the model included polygenic effects.

Model BayesCπ without polygenic effects was applied to obtain the GEBVs of the final generation (5), with training on the first four generations because it resulted in high accuracy and small bias of GEBV based on training in the first three generations. Results at the bottom of Table 1 show that the GEBVs from training on the first four generations were more accurate and less biased compared with training on the first three generations, because the training population size increased by 977 individuals and the SNP effects were more accurately estimated.

### Estimated variances

Variance components estimated by the different models are shown in Table 2. Including polygenic effects in the model resulted in a larger estimated genetic variance and a smaller residual variance, and the estimated heritability was closer to the true value of 0.5, in accord with Calus *et al.*[18]. However, since no polygenic effects were simulated in the QTLMAS2010 dataset, including polygenic effects underestimated the variance explained by the SNPs, because some genetic variance due to relationships captured by the SNPs was taken over by polygenic effects. Furthermore, the estimated variance components were not sensitive to the average number of SNPs included in the model, showing that around 100 SNPs were sufficient to capture most genetic variance.

### QTL Mapping

**Figure 1:**
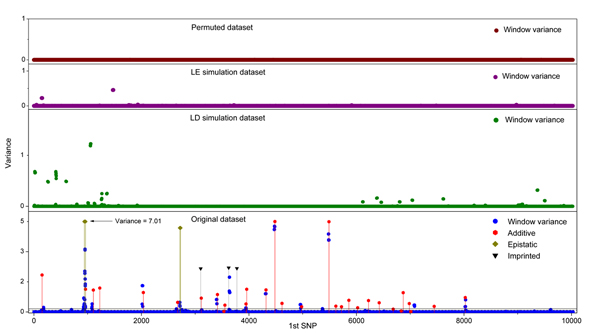
**Variances of GEBVs of 10-SNP windows across the genome.** Data sets were generated by permutation (Permuted dataset), simulation with linkage equilibrium in founders (LE simulation dataset), and simulation with initial linkage disequilibrium (LD simulation dataset). The bottom panel show window variances obtained for the original QTLMAS 2010 dataset (Original dataset), as well as the location and variances of true QTLs, along with their mode of inheritance (Additive = additive QTL, Epistatic = epistatic QTL, Imprinted = imprinted QTL). Horizontal lines show the 10% (solid) and 20% (dash) chromosome-wise thresholds for window variance derived from the LD simulation.

Several parameters estimated by BayesCπ can be used to identify QTL regions, for instance, the absolute estimated effects of SNPs, the posterior inclusion probabilities (model frequencies) of SNPs, and the genetic variances explained by SNPs. Many Bayesian QTL mapping studies have applied model frequency or its derivatives as criteria to detect QTL [7-9]. In those studies the markers were less dense and QTL were expected to be in LD with only one or several adjacent markers. However, for the high density SNP panel of the QTLMAS2010 data, the QTL and markers are expected to be in high LD (average *r*^2^=0.22 between adjacent markers on chromosome 1) and the effect of a single QTL could be spread over multiple SNPs. This results in too many signals in model frequency which could increase the probability of false positives and false negatives. To address this problem, we accumulated the effects of adjacent SNPs together into a genomic window. A window size of 10 was used in this study and the variance of GEBV of each 10-SNP window was used as the criterion to detect QTL. Several windows that shared the same SNP with a large effect were considered to identify the same QTL region. Within each region, because windows were overlapping, the window with the highest variance of GEBV was used and the SNP within this window that explained the largest proportion of genetic variance was used to denote the position of the QTL (Figure 1).

Results of the three strategies to set significant thresholds are summarized in Table 3. Plots of window variances against the identity of the first SNP of each window are shown in Figure 1. With permutation of phenotypes against genotypes, the SNPs did not capture much genetic variance because pedigree relationships were destroyed and the variances of all 10-SNP windows were close to zero. Consequently the thresholds set by permutation were extremely low. The threshold determined by simulation of SNP genotypes was more reasonable than that from permutation because the relationships between individuals remained unchanged. Because no QTL existed in the simulated genotypes, the SNPs only captured pedigree relationships. Genotypes of SNPs simulated without and with linkage disequilibrium in the founders captured similar proportions of total variance, but different subsets of SNPs were fitted in the model. The average number of SNPs in the model in the MCMC iterations was similar with 124 SNPs for simulated data sets due to the strong fixed prior π, but the model frequencies of the fitted SNPs were higher when LD was simulated and each of these SNPs explained a more genetic variance. As a result, the window variance thresholds were much higher for the data set with LD among founders. Therefore, for QTLMAS2010 data, where the genetic relationship among individuals were known, pedigree-based simulation of genotypes with initial LD was used to obtain the distribution of window variances under the null hypothesis of no intrinsic relationships between marker genotypes and phenotypes.

**Table 3:** Variance components estimated from datasets generated by permutation, simulation with linkage equilibrium in founders (LE simulation), and simulation with initial linkage disequilibrium (LD simulation), and thresholds for 10-SNP window variances based on 10% and 20% chromosome-wise type I error rates.

Methods	Variance Components			Window variance threshold	
	
	Genotypic	Residual	Total	10%	20%
Permutation	3.15	98.59	101.74	0.0011	0.0009
LE simulation	20.83	79.84	100.67	0.0204	0.0094
LD simulation	17.14	83.40	100.55	0.1645	0.0887
Original^1^	47.13	53.48	100.61	–	–

^1^Estimated from the original QTLMAS2010 dataset using BayesCπ, training on the first 4 generations.

The threshold allowing a 10% chromosome-wise type-I error rate detected 13 QTLs of which 2 were false positives (Figure 1). Each of the epistatic QTL-pairs was detected as one large QTL. A total of 20 small additive QTLs and 2 imprinted QTLs were missed. The threshold allowing a 20% chromosome-wise type-I error rate identified 6 more QTLs but 4 of these were false positives.

Adjustment for multiple testing was based on a Bonferroni-type of adjustment based on an estimate of the effective number of independent tests conducted. A more appropriate adjustment for multiple testing would be replicating the simulation multiple times and picking the highest window variance within each simulation. This replication procedure would resemble the method based on permutation tests proposed by Churchill *et al*. [10], but would be more expensive computationally.

The window variance calculated using the sum of model-averaged SNP effects within a specific window will always underestimate the true QTL variance because of the shrinkage of SNP effects by BayesCπ and the incomplete LD between SNPs and QTL. Estimation of the variance of a window can be improved by computing the variance based on the sampled window effects from each sample of the MCMC chain, which is less shrunk than the posterior mean of the window effects.

Although grouping SNPs into windows is effective to concentrate signals, it also has several drawbacks. First, if say two QTL fall into the same region, by window variance they would likely be detected as one QTL; for example, additive QTL11 and QTL12 were detected as a single QTL (Figure 1). Second, the effect of a single QTL may spread over more markers than the window length, especially in regions with weak LD between QTL and SNP; in this case windows over a wide region may show high variance, giving rise to the detection of multiple QTL for a region in which there is only one QTL. This is very likely to be the reason for the false positives reported around QTL1 (Figure 1), whose effect spread over more than 40 SNPs when estimated by BayesCπ. Third, window variance works well for relatively large QTL, but may shrink signals for small QTL, such as the eight undiscovered QTL on chromosome 4. Most of these eight QTL had detectable signals of SNP model frequency, but the window variances were below the thresholds that were set. All these drawbacks need to be further investigated, including the optimal size of windows to use.

The use of windows in this study is fundamentally different from the use of haplotypes to detect QTL, although both use combinations of adjacent markers. An alternative method may well be constructing haplotypes using two or more adjacent SNP alleles and estimating haplotype effects using Bayesian methods. Villumsen *et al.*[19] showed that there is an optimal haplotype length for the accuracy of GEBV prediction depending on the population, LD, and marker spacing. Using haplotypes allows combining linkage disequilibrium and linkage analysis information by including the probability of identity-by-decent between haplotypes at the same locus, and the improved accuracies of LD-mapping [20] and genomic selection [21] have already been reported. It is hence worthwhile to investigate the use of haplotype on the precision of Bayesian QTL mapping.

## Conclusions

In this simulated dataset, BayesCπ slightly outperformed BayesB in the accuracy of predicting GEBV, but the accuracy of BayesB was similar to BayesCπ when its π was set equal to the posterior mean of π from BayesCπ. The prediction accuracy of TBV was lower than that of genotypic values. Window variance allowed detection of most large QTLs but had insufficient power to detect the small QTLs. Since the model only captured additive effects of QTLs, each epistatic QTL-pair was detected as one multi-allelic additive QTL and the two imprinted QTLs were not detected. The results expose the need for advanced statistical approaches to address more complicated patterns of genetic effects that exist in real data.

## List of abbreviations used

GEBV: Genomic Estimated Breeding Value; SNP: Single Nucleotide Polymorphism; QTL: Quantitative Trait locus; LD: Linkage Disequilibrium; GWAS: Genome-wide Association Study; BLUP: Best Linear Unbiased Prediction; EBV: Estimated Breeding Value; MCMC: Markov Chain Monte Carlo; LE: Linkage Equilibrium; TBV: True Breeding Value

## Authors’ contributions

XS carried out the analysis and drafted the manuscript. DH contributed to programming BayesCπ, developed the program for pedigree-based simulation, and helped to interpret the results. RLF contributed to programming BayesCπ and helped to interpret the results. DJG contributed to programming BayesCπ and helped to interpret the results. JCMD was the overall coordinator of the project, developed the method to set thresholds, and helped to interpret the results and draft the manuscript.

## Competing interests

The authors declare that they have no competing interests.
